# Editorial: Cell-Based Therapies for Diabetic Microvascular Complications

**DOI:** 10.3389/fendo.2015.00146

**Published:** 2015-09-22

**Authors:** Ashay D. Bhatwadekar

**Affiliations:** ^1^Eugene and Marilyn Glick Eye Institute, Indianapolis, IN, USA

**Keywords:** editorial, diabetic microvascular complications, diabetes, cell-based therapies, adult stem cells, progenitor cells

There is an alarming increase in the number of cases of diabetes with the global prevalence rate at 9% for the year 2014 (1). Around 387 million patients worldwide are suffering from diabetes, and this number is expected to increase by 50% by 2030 (2). In particular, the South East Asian countries are affected most due to diabetes with projected 10% increase in diabetic patients by 2035 (3). Diabetes is strongly associated with microvascular complications and with the rise in the numbers of people with diabetes, there is a precipitous increase in patients suffering from diabetic microvascular complications (4, 5). Most of the microvascular beds are affected in diabetes but those in the kidney, retina, and myelinated nerves get affected the most. In diabetes, there is a constant demand of new cells to “replace” the dying endothelium and to keep the microvasculature healthy in order to meet the physiological demands of respective tissues (5). However, metabolic perturbations of diabetes fail to maintain the burgeoning demand of dying endothelium creating widespread areas of ischemia and underperfusion. While the pharmaceutical development of anti-diabetic medications is mainly centered on controlling hyperglycemia, there is an apparent lack of therapies which specifically are focused on promoting the endogenous repair of the vasculature. Studies over the past several years highlight that cell-based therapies involving adult stem and progenitor cells hold promise of rescuing the dying endothelium in diabetes. This novel therapeutic strategy involves stem cells obtained from a variety of sources, such as circulating endothelial progenitor cells, mesenchymal stromal cells (MSCs), embryonic stem cells, and inducible pluripotent stem cells (iPSCs). This research topic discussed therapeutic potential and recent developments in cells therapies for the treatment of diabetic microvascular complications.

Mesenchymal stromal cells are at a forefront of the cell-based therapies and Davey et al. elegantly summarized importance of MSCs for diabetic microvascular complications (6). The review article suggests that MSCs play a critical role in the treatment of microvascular complications due to the multipotency and paracrine mechanisms of MSCs. The treatment of MSCs is reported to help in maintaining glycemic control by differentiation of MSCs into insulin-producing cells (7). MSC injection in animal model of spinal cord injury results in local upregulation of neurotrophic factors, such as nerve growth factor (NGF), and restoration of nerve conduction velocity (8). Using an animal model of diabetic nephropathy, previous studies have shown engraftment of 11% of MSCs into the kidneys of diabetic animals. MSC treatment resulted in a decrease in mesangial thickening and macrophage infiltration, thus helping to correct kidney dysfunction in diabetes (9). In addition to diabetic microvascular complications, the MSCs are also shown to be beneficial in the treatment of diabetic wound healing (10), cardiomyopathy (11), and bone-fracture (12). It is noteworthy that there are about 14 open clinical trials using MSCs to treat the diabetes and associated complications.

Dr. Rajashekhar further reiterated the importance of MSCs in diabetic microvascular complications in his review by summarizing the critical benefits of MSCs for the treatment of diabetic retinopathy (13). His article suggests that MSCs derived from adipose tissues [i.e., adipose-derived stem cells (ASCs)] possess similarities with pericytes. Retinal pericytes provide necessary support for the retinal vasculature. Diabetic retinopathy is associated with the loss of pericytes (14). His studies suggest that ASCs help in the treatment of injured retina either by paracrine repair or by physical proximity with the endothelial cells (15). Adipose tissue is the primary source of ASCs which provides an advantage of ease of isolation and abundance as compared to bone marrow.

Furthermore, Mizukami and Yagihashi summarized the importance of ASCs in the treatment of diabetic neuropathy (16) adding to Dr. Rajashekhar’s research on diabetic retinopathy. Their review suggests that ASC treatment releases neurotrophic factors such as epidermal growth factor, transforming growth factor-β1 (TGF-β1) (17), vascular endothelial growth factor (VEGF), basic fibroblast growth factor (bFGF), hepatocyte growth factor, insulin like growth factor 1 (IGF-I), and brain-derived neurotrophic factor (BDNF) while exhibiting a reparative function in diabetic neuropathy (18). In addition to the release of growth factors, the ASCs also differentiate into target organ in small percentage and possess immunosuppressive effects (19).

Adding to the novel stem cell therapies for diabetic microvascular complications, Lois et al. summarized the importance of endothelial cell forming cells (ECFCs) in diabetic retinopathy (20). Their studies suggest that ECFCs possess a remarkably high proliferative capacity and tend to integrate into mature endothelial cells (21). These ECFCs when injected *in vivo* home to ischemic retina and integrate into retinal vasculature (22).

While stem cell treatments for diabetic mircrovascular complications are promising, diabetes leads to defects in their normal reparative function. Our studies suggest that diabetes results in dysfunction of CD34^+^ cell (23) of diabetic patients. Diabetic CD34^+^ cells show decrease in migration, proliferation, and incorporation (24) in blood vessels. We characterized that defects in diabetic CD34^+^ cells are observed due to decrease in nitric oxide (NO) levels and restoration of NO helps in correcting CD34 dysfunction (23). We used a variety of pharmacological agents like TGF-β1 morpholino (25), angiotensin 1-7 (Ang 1-7) (26) to correct low levels of NO and restore the functional ability of diabetic CD34^+^ cells.

In the era of regenerative medicine, stem cell treatments for diabetic microvascular complications hold a significant potential; however, there are series of challenges to overcome such as (i) choice of an ideal stem cell type, (ii) rigorous characterization of stem cells, (iii) site of injection and route of delivery, (iv) limited survival, (v) risk of tumors, and (vi) impaired potency, before the cell therapy reaches to the clinic. However, with advancement in research, stem cells will provide effective treatments for diabetic microvascular complications.

## Conflict of Interest Statement

The author declares that the research was conducted in the absence of any commercial or financial relationships that could be construed as a potential conflict of interest.
